# Treatment of COVID-19 olfactory dysfunction with olfactory training, palmitoylethanolamide with luteolin, or combined therapy: a blinded controlled multicenter randomized trial

**DOI:** 10.1007/s00405-023-08085-8

**Published:** 2023-06-28

**Authors:** Arianna Di Stadio, Salvatore Gallina, Salvatore Cocuzza, Pietro De Luca, Angelo Ingrassia, Simone Oliva, Federico Sireci, Angelo Camaioni, Fabio Ferreli, Giuseppe Mercante, Francesca Gaino, Gian Marco Pace, Ignazio La Mantia, Michael J. Brenner

**Affiliations:** 1 Otolaryngology Unit, GF Ingrassia Department, University of Catania, Catania, Italy; 2 Otolaryngology Department, University of Palermo, Palermo, Italy; 3 Otolaryngology Department, San Giovanni-Addolorata Hospital, Rome, Italy; 4 Otolaryngology Department, Humanitas University Hospital, Milan, Italy; 5 Department of Otolaryngology-Head and Neck Surgery, University of Michigan Medical School, Ann Arbor, MI USA

**Keywords:** Anosmia, COVID-19, SARS-CoV-2, Coronavirus, Neuroinflammation, Olfactory training, PEA, PEA–LUT, Smell disorders, Palmitoylethanolamide, Luteolin, Randomized trial, Clinical trial, Post-acute sequelae of COVID-19, Hyposmia

## Abstract

**Purpose:**

Few evidence-based therapies are available for chronic olfactory dysfunction after COVID-19. This study investigated the relative efficacy of olfactory training alone, co-ultramicronized palmitoylethanolamide with luteolin (um-PEA–LUT, an anti-neuroinflammatory supplement) alone, or combined therapy for treating chronic olfactory dysfunction from COVID-19.

**Methods:**

This double-blinded controlled, placebo-controlled multicenter randomized clinical trial was conducted in 202 patients with persistent COVID-19 olfactory dysfunction of > 6 month duration. After a screening nasal endoscopy, patients were randomized to: (1) olfactory training and placebo; (2) once daily um-PEA–LUT alone; (3) twice daily um-PEA–LUT alone; or (4) combination of once daily um-PEA–LUT with olfactory training. Olfactory testing (Sniffin’ Sticks odor identification test) was performed at baseline and at 1, 2, and 3 months. The primary outcome was recovery of over three points on olfactory testing, with outcomes compared at *T*_0_, *T*_1_, *T*_2_ and *T*_3_ across groups. Statistical analyses included one-way ANOVA for numeric data and chi-square for nominal data.

**Results:**

All patients completed the study, and there were no adverse events. At 90 days, odor identification scores improved by > 3 points in 89.2% of patients receiving combined therapy vs. 36.8% receiving olfactory training with placebo, 40% receiving twice daily um-PEA–LUT alone, and 41.6% receiving once daily um-PEA–LUT alone (*p* < 0.00001). Patients receiving treatment with um-PEA–LUT alone demonstrated subclinical improvement (< 3 point odor identification improvement) more often than patients receiving olfactory training with placebo (*p* < 0.0001.)

**Conclusions:**

Olfactory training plus once daily um-PEA–LUT resulted in greater olfactory recovery than either therapy alone in patients with long-term olfactory function due to COVID-19.

**Trial registration:**

20112020PGFN on clinicaltrials.gov.

**Level of evidence:**

1b (Individual Randomized Clinical Trial).

## Introduction

Transient anosmia is often present in the acute phase of Severe Acute Respiratory Syndrome Coronavirus 2 (SARS-CoV-2) infection, and it occurs most commonly in patients with mild or moderate COVID-19 [1–4]. Although the pathogenesis of COVID-19 olfactory dysfunction has not been fully elucidated, neuroinflammation is thought to play a critical role [5]. Susceptibility is likely multifactorial, influenced by the viral load at initial exposure, the SARS-CoV-2 variant involved, and patient factors, such as overall health status or genetic predisposition to neuroinflammation [6]. Most individuals who experience olfactory or gustatory loss spontaneously recover baseline function within a few weeks [1], but others manifest impaired smell or taste long after the acute phase of illness has subsided [3, 4]. This persistent sensory loss has implications for safety (detection of gas, smoke, or toxins) and quality of life, adversely affecting not only subjective enjoyment of food and aromas, but also appetite, nutrition, and overall well-being [7]. Olfactory loss is a common feature of the post-acute sequelae of SARS CoV-2 infection (PASC), often described as long COVID [8].

The unmet needs of patients with long COVID and olfactory loss represent a looming public health crisis [9]. Although several treatments have been investigated [10, 11], most treatments have yielded limited or no benefit. In the pre-COVID era, the only therapy with high level evidence of efficacy for treating post-viral olfactory loss was olfactory training [12]. The pandemic provided an impetus for exploring new therapies, however. Co-ultramicronized (um) Palmitoylethanolamide (PEA) and luteolin (LUT) (um-PEA–LUT) has been used in treatment of neuroinflammatory and neurodegenerative diseases, such as multiple sclerosis and dementia. This supplement contains PEA, a molecule normally produced by microglia cells (specialized immune cells found in the central nervous system that contribute to neuroprotection and regulation of inflammation) and luteolin, a flavonoid extract known for its antioxidant and anti-inflammatory properties [13].

In the pandemic era, um-PEA–LUT has been repurposed as a potential therapy of COVID-19 olfactory impairment. The PEA component of the supplement acts on microglia to promote an anti-inflammatory, neuroprotective and tissue repair profile (M2 phenotype). The LUT component reduces reactive oxygen species (ROS), and in patients with persistent olfactory loss after COVID-19, this combination therapy is thought to counteract inflammation of olfactory bulbs and neuro-epithelium. A prior randomized trial found that the combination of um-PEA–LUT and olfactory training allowed better olfactory recovery than olfactory training alone [13]. This regimen has also ameliorated memory impairment in patients who reported brain fog [14]; in these studies, olfactory recovery correlated with improvements in memory [1]. However, all the previously conducted studies used the minimal dose of um-PEA–LUT, because the preliminary endpoint assessed if the supplement had an efficacy in treating persistent post‐COVID‐19 olfactory dysfunction. Optimal dosing and comparisons with monotherapy regimens have not been investigated.

These data provided the impetus for the present study. Neuroinflammation has been implicated in the pathogenesis of post-viral smell disorders [15], providing the rationale for treating chronic COVID-19 olfactory dysfunction with um-PEA–LUT. Neuroinflammation has also been linked to other clinical features of long COVID, including cough [16], psychiatric symptoms, or neurologic impairment [17]. Um-PEA–LUT therapy, which reduces neuroinflammation, is, therefore, a candidate therapy to alleviate disorders of smell, cognition, and other post-acute sequelae of SARS CoV-2 infection. To date, this therapy has been studied only when combined with olfactory training. Although the prior studies yielded promising results, use of the supplement alone or at different dosages has not been studied. When used for treating neuro-inflammation (Alzheimer Disease or Multiple Sclerosis), um-PEA–LUT may be administered in once daily or twice daily regimens. Neurologists often start with two sachets per day for 30–40 days, reducing to once daily for 6 months.

This double-blinded multicenter clinical trial investigated four therapeutic regimens for persistent post-COVID-19 olfactory dysfunction. It sought to determine (1) the relative efficacy of um-PEA–LUT alone, olfactory training alone, and combined therapy with um-PEA–LUT plus olfactory training (OT) for promoting olfactory recovery; (2) whether olfactory recovery differed among patients receiving once-daily vs. twice-daily administration of um-PEA–LUT alone.

## Materials and methods

### IRB approvals and general considerations

**Fig. 1:**
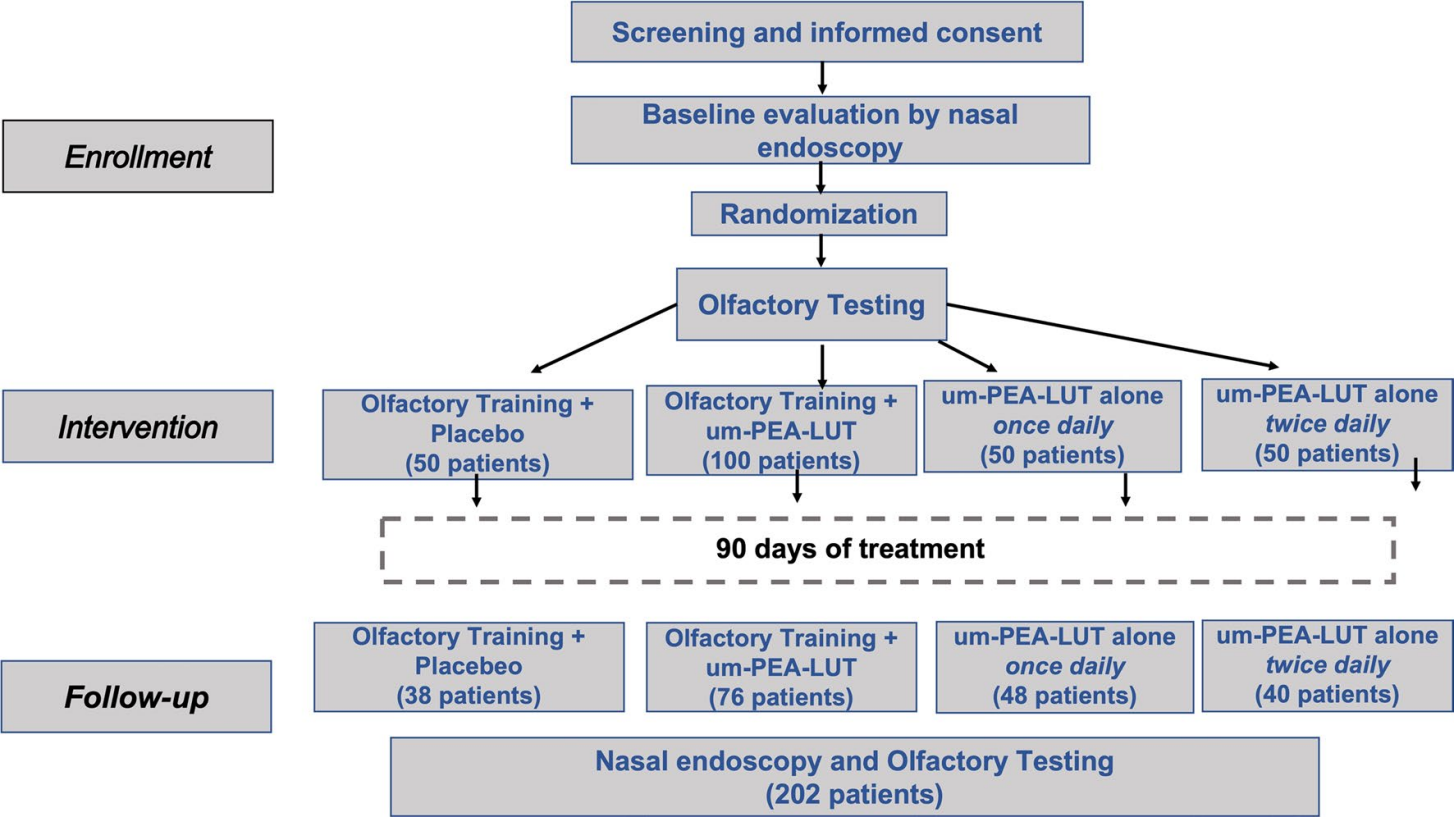
Experimental design and randomization scheme with allocation of the patients across the 4 different groups. Allocation reflects double weighting in randomization for the putative gold standard (olfactory training + um-PEA–LUT). *um-PEA–LUT* ultramicronized palmitoylethanolamide with luteolin

This study adhered to CONSORT guidelines for Clinical Trials (Fig. 1). We performed a multi-center double-controlled-blinded, placebo-controlled clinical trial. All patients recruited to the study underwent counseling, screening, treatment, and assessment in the otolaryngology clinics of participating centers. The study protocol was approved by the Institutional Review Board of Humanitas University (identifier 3002) and was registered at Clinicaltrials.gov in April 2021 with number: 20112020PGFN. All aspects of the study were conducted in strict accordance with the Declaration of Helsinki. All patients signed a written consent prior to inclusion in the study.

### Study population and demographic data

This trial was open to patients at three referral hospitals from November 2021 to August 2022. To recruit patients, we used word of mouth communication among clinicians and calls through newspaper, television, and internet. All centers adhered to the same procedures and protocols. Patients were included or excluded based on the following criteria:

#### Inclusion criteria

Eligible participants for this study included outpatients, ages 18–60 years, with a confirmed history of COVID-19 (verified by positive nasopharyngeal swab for SARS-CoV-2, analyzed by RT-PCR testing), and presence of persistent anosmia or persistent hyposmia. Olfactory dysfunction was evaluated based on a reduced odor identification score using the Sniffin’ Sticks psychophysical test. Persistent olfactory dysfunction was defined as an odor identification score < 14 that persisted for > 180 days (6 months) after a subsequent negative COVID-19 nasopharyngeal swab. Prior to taking part in the study, individuals were counseled regarding the study, including the option to decline or withdraw at any time.

#### Exclusion criteria

Individuals were excluded from the study if they had a previous history of olfactory or gustatory disorders, were undergoing active chemotherapy, radiation therapy, or treatment with estrogen inhibitors (aromatase), had impaired baseline cognitive function, had a history of neurodegenerative disease (Alzheimer, Parkinson, or Lewy body Disease), or were receiving medical therapy with known risk of affecting olfactory function. Additional exclusion criteria included presence of rhinological disorders (sinusitis, rhinosinusitis, sinonasal polyposis, atrophic rhinitis, allergy) at time of enrollment, history or chemo-radiotherapy of the head and neck region, history of stroke or neurotrauma, nasal blockage from stenosis or deformity, severe psychiatric illness (e.g., schizophrenia, bipolar disorder, olfactory hallucination), previous sinonasal or nasopharyngeal tumors, or use of corticosteroid therapy to treat olfactory dysfunction within the past 30 days. In addition, any patients requiring maintenance on medications with anti-inflammatory or immune-modulating effects or who were not interested in participating were excluded from the study.

### Demographic data extraction

For each patient, we collected following demographic data: sex, age, medical disorders, tobacco, alcohol, or other substance use, medications, prior history of olfactory dysfunction, prior treatment for COVID-19-associated olfactory disorders, and time elapsed since negative COVID-19 test. We created a medical record for each patient that included: the patient’s general medical and family health history, details on COVID-19 illness (date and symptoms at onset, date of positive and negative PCR-based SARS-CoV-2 testing, treatments during the infection, persistent symptoms, and treatments used after COVID-19 resolution), and data on COVID-19 vaccination. We also recorded detailed data on smell and taste alterations. After verbally explaining the differences between anosmia, hyposmia, parosmia, and phantosmia to patients and clarifying the difference between taste alteration and sense of smell/flavor, we queried patients about their history. At the end of the study (3 months after randomization and initiation of therapy), data were extracted and analyzed by a statistician, following procedures stipulated by the study coordinator (ADS).

### Experimental groups

The four study groups were defined as follows:

1. Combined therapy (um-PEA–LUT: once daily + olfactory training): These patients received daily treatment with one sachet of orally administered co-ultra-micronized Palmitoylethanolamide 700 mg and Luteolin 70 mg (Glialia^®^, Epitech Group SpA, Milano, Italy), (um-PEA–LUT)**,** which was administered as a single dose, 5–10 min before breakfast plus olfactory training. Olfactory training entailed stimulation using four organic essences (Lemon, Rose, Eucalyptus and Cloves) administered three times every day for 6 min each session; stimulation consisted of smelling an odor for 4–6 s, then 40 s of relaxation, and then new stimulation for 4–6 s with another essence. This short duration was used to avoid saturation of the olfactory receptors [6, 13, 18]. Subjects performed this regimen for 90 consecutive days.
2. Um-PEA–LUT alone (once daily): These patients received once daily treatment with one sachet of um-PEA–LUT, administered 5–10 min before breakfast for 90 days.
3. Um-PEA–LUT alone (twice daily): These patients received twice daily treatment with one sachet of um-PEA–LUT administered 5–10 min before breakfast and one sachet of um-PEA–LUT administered 5–10 min before lunch for 90 days.
4. Olfactory Training plus placebo supplement (control): These patients received olfactory training using the same regimen as in the combined therapy group above plus a daily placebo supplement therapy (generic multivitamin).

All patients performed the olfactory training independently at home after receiving in-person detailed education and practical training, as overseen by the physician. The initial education consisted of a face-to-face explanation on how to perform olfactory training followed by practice performing the exercise until competency was verified. In addition, a written description on how to prepare the essences was provided. This instruction was reinforced by providing access to an instructional video, which provides detailed instruction on how to perform the basic steps of olfactory training (https://www.youtube.com/watch?v=Ri5YwM6EmWM). This video resource and all other aspects of the study were kept consistent across all patients and sites, and there was ongoing engagement by the study team to assess for adherence and adverse effects. All patients underwent nasal endoscopy before the Sniffin’ sticks assessment. Participants in the study were in frequent communication with clinic staff and physicians to promote adherence to the study protocol, involving 1 contact every 2 weeks. These follow-up interactions were accomplished via phone calls, electronic health record communications, video visits, and in-person office visits.

All patients (including the control group and the three therapeutic intervention groups) underwent the following nasal endoscopic examination at *T*_0_ (baseline) and *T*_3_ (90 days after treatment). The *T*_0_ nasal endoscopic examination evaluated for presence of polyps, masses, anatomic blockage, or other pathology, any of which would result in exclusion from the study. All patients were evaluated for olfactory function using Sniffin’ Sticks (Burghardt^®^, Medisense, Winschoten, The Netherlands). This initial olfactory evaluation was performed at the outset of the study (*T*_0_). Patients were subsequently re-evaluated by Sniffin’ Sticks assessment every 30 days, corresponding to observation points: *T*_0_ (baseline), *T*_1_ (30 days), *T*_2_ (60 days), and *T*_3_ (90 days; endpoint).

### Randomization and blinding

Patients were assigned a number at time of recruitment, and this reference number was used for tracking during randomization and throughout the study protocol. Using a block randomization within each site, eligible participants were randomly assigned [19, 20]. Randomization involved double allocation to the combined therapy (um-PEA–LUT plus OT), which aimed to maximize exposure to the evidence-based treatment regimen and to facilitate detection of any adverse effects [21–26]. After patient counseling and consent, the physician used computer-generated randomization for assignment of patients. This randomization scheme was based on prior work supporting combined therapy (um-PEA–LUT plus olfactory training) as effective therapy for persistent olfactory loss. Double weighting of the combined group was operationalized by duplicating the combined therapy group, thereby doubling the predicted enrollment to this experimental arm, similar to prior experimental design [13].

Patients were informed that the purpose of the study was to investigate approaches for treating persistent loss of smell after COVID-19 and were provided information on the proposed regimen. Patients were informed that after baseline assessment, they would be randomized to a treatment involving taking an experimental supplement or placebo, with or without olfactory training. Patients were counseled that the supplement had no reported drug interactions or known safety-related concerns. Patients were also counseled regarding the placebo-controlled design of the study with blinding regarding supplement. They were informed that after baseline assessment, they would repeat the Sniffin’ Sticks assessment at 30, 60 and 90 days. Patients were counseled that their participation was voluntary and that they could withdraw from the study at any time.

The study was performed in a double-blinded, placebo-controlled manner. The patients did not know their status with respect to placebo or um-PEA–LUT supplement (patient-blinding), although they were not blinded as to performing olfactory training. A single physician at each center performed the nasal endoscopy, and a second physician performed olfactory testing. The physician who performed olfactory testing remained blinded to experimental group throughout the study. The physician who performed the nasal endoscopy had knowledge of the experimental groups and did not participate in olfactory assessment. Having physician roles thus separated ensured that individuals performing assessments of olfactory recovery were always blinded to the patients’ treatment arm. Data obtained by the olfactory test were anonymized and results were collected on a protected Excel sheet shared by all the centers [Google (Mountain View, California, USA)].

### Assessment of olfactory dysfunction

The Sniffin’ Sticks identification test was administered to assess olfactory function following a previously established protocol [27]. Briefly, clinicians used standard pen-like devices filled with odorants to score olfactory function and were blinded to the patients’ experimental groups. During the odor identification task, participants were presented with 16 common odors (Blue set of Sniffin’ stick). In multiple-choice format, participants were asked to select which of 4 odor labels matched the presented odor. Possible scores for odor identification ranged from 1 to 16. These scores were used to classify olfactory function as anosmia (score < 7), hyposmia (score from 7 to 13), or normosmia (score ≥ 14), and scores were then recorded for subsequent analysis. We used the Sniffin Sticks test, because based on prior experience and ability to provide facile comparisons to prior work using both the complete the complete test (Thresholds, Detection and Identification) [6, 13, 19] and the Identification (I) test only [14]. The Identification (I) test was used in isolation to minimize fatigue and promote attention, as the extended version of this olfactory test and UPSIT tests might reduce patients’ adherence in this long Covid cohort [13]. When we compared identification scores at follow-up intervals, an increase of three points was considered significant, as previously described [28, 29].

Using the full TDI battery allow for a more comprehensive standard; however, the threshold of a 5-point change used for the full TDI battery (10% change relative to the maximal possible score of 48), is potentially less stringent than the 3-point change required when assessing differences with Identification alone, (19% change relative to the maximal possible score of 16) [29].

### Sample size calculation

The sample size was calculated as described by Wang and Ji [26], utilizing the protocol specific for Randomized Clinical Trials available at calculator.net (https://www.calculator.net/sample-size-calculator.html?type=1&cl=95&ci=6.5&pp=50&ps=&x=43&y=8), with design incorporating a 95% confidence interval and < 7% margin of error.

### Statistical analyses

One-way ANOVA for repeated measures and Bonferroni–Holmes (BH) post-hoc tests were used to analyze statistical differences in Sniffin’ Sticks score within each experimental group comparing *T*_0_, *T*_1,_ *T*_2_, and *T*_3_, and verification that data were normally distributed. The same statistical procedures were used for between-group comparisons. Patients were classified as having subclinical recovery (< 3 points), clinically significant recovery (≥ 3 points), unchanged (0-point change), or worsened (≥ 1 point decrement). The number of patients in each category was assessed within and between groups over the course of the study, analyzed by Chi-square (*χ*). Statistical significance was set at *p* < 0.05 with two-tailed test. All analyses were performed using Stata^®^ and performed by a statistician who was blinded to the treatments performed by each group.

#### Role of the funding source

There was no funding source for this study.

## Results

### General

From 250 patients enrolled, a total of 202 patients (80.8%) completed the study, performing assessments at *T*_1_, *T*_2_ and *T*_3_. Individuals who did not complete all 3 timepoints were excluded from the study. None of the subjects reported suffering from parosmia and/or mental clouding (brain fog). The gold standard, (olfactory training + um-PEA–LUT, based on prior studies), was double-weighted in randomization, as described in methods, resulting in these patient assignments:

38 patients (26 women and 12 men, age average 40.9 ± 11.7) in olfactory training + placebo group; 48 patients (18 women and 30 men, age average 39.8 ± 11.5) in the um-PEA–LUT once daily group; 40 patients (24 women and 16 men, age average 37.1 ± 13.9) in the um-PEA–LUT twice daily group; 76 patients (40 women and 36 men, age average 42.7 ± 13.5) in olfactory training + um-PEA–LUT.

The rate of attrition for this study was 19.2% (48 of the 250 participants recruited to the study). This attrition by group was 12/50 (24%) for the OT + placebo group, 2/50 (4%) for the once daily um-PEA–LUT group, 10/50 (20%) for the twice daily um-PEA–LUT alone group, and 24/100 (24%) for OT + um-PEA–LUT group. Chi square analysis demonstrated that the attrition rate was lower in the once daily um-PEA–LUT group than in the other three groups (*p* < 0.001). In analyses comparing baseline characteristics of randomized participants who completed the study to those who dropped out of the study, men were more likely than women to drop out of the study (*p* = 0.01). There were no differences in baseline (*T*_0_) Sniffin’ Sticks score, in Sniffin’ Sticks score change over time at *T*_1_ and *T*_2_, or other sociodemographic parameters based on participant dropout vs. study completion.

Table 1 shows characteristics of each group, including comorbidities. Table 2 shows treatments that patients used in the past for treating olfactory disorders.

**Table 1:** Details about the characteristics of each group, including comorbities

Characteristics	Number (%)			
	Control	PEA–LUT + OT	PEA–LUT × 1	PEA–LUT × 2
Age, mean (SD)	40.9 ± 11.7	42.7 ± 13.5	39.8 ± 11.5	37.1 ± 13.9
Sex	Number (%)			
Women	26 (68.4%)	40 (71.4%)	18 (37.5%)	24 (60%)
Men	12 (31.6%)	16 (28.6%)	30 (62.5%)	16 (40%)
	Mean SD			
Identification score (baseline)	6.5 ± 3.7	8.2 ± 2.5	9.1 ± 3.4	9.6 ± 4.1
Months of affection	6.8 ± 2.6	8.8 ± 3.4	6.8 ± 3.5	11 ± 1.2
Comorbidities	Number (%)			
Smoke	2 (5%)	4 (7.6%)	2 (4.1%)	8 (20%)
Heart disease	2 (5%)	2 (3.5%)	2 (4.1%)	4 (10%)
Hypertension	0	2 (3.5%)	2 (4.1%)	2 (5%)
diabetes	0	0	0	0
Neurologic disorder	0	2 (3.5%) headache	0	1 (2.5%) epilepsy
Psychiatric disease	0	0	0	0

*SD* standard deviation, *PEA–LUT + OT* supplement 1 dose day and olfactory training, *PEA–LUT × 1* supplement 1 dose day, *PEA–LUT × 2* supplement two dose day, *PEA–LUT* ultramicronized palmitoylethanolamide with luteolin, *OT* olfactory training, *SD* standard deviation

**Table 2:** Treatments that patients used in the past for treating the smell disorders and that were suspended at least 40 days before the enrollment in the study

	Number (%)			
Previous treatment	Control	PEA–LUT + OT	PEA–LUT × 1	PEA–LUT × 2
Steroid	2 (5%)	26 (46.4%)	6 (12.5%)	16 (40%)
Vitamins	2 (5%)	12 (21.4%)	2 (4.1%)	8 (20%)
Alfa-lipoic/nicetil/other	2 (5%)	10 (17.8%)	2 (4.1%)	16 (40%)

*PEA–LUT + OT* supplement 1 dose day and olfactory training, *PEA–LUT × 1*: supplement 1 dose day, *PEA–LUT × 2*: supplement two dose day, *PEA–LUT* ultramicronized palmitoylethanolamide with luteolin, *OT* olfactory training

### Primary outcome: between groups comparison, clinically meaningful olfactory recovery

**Fig. 2:**
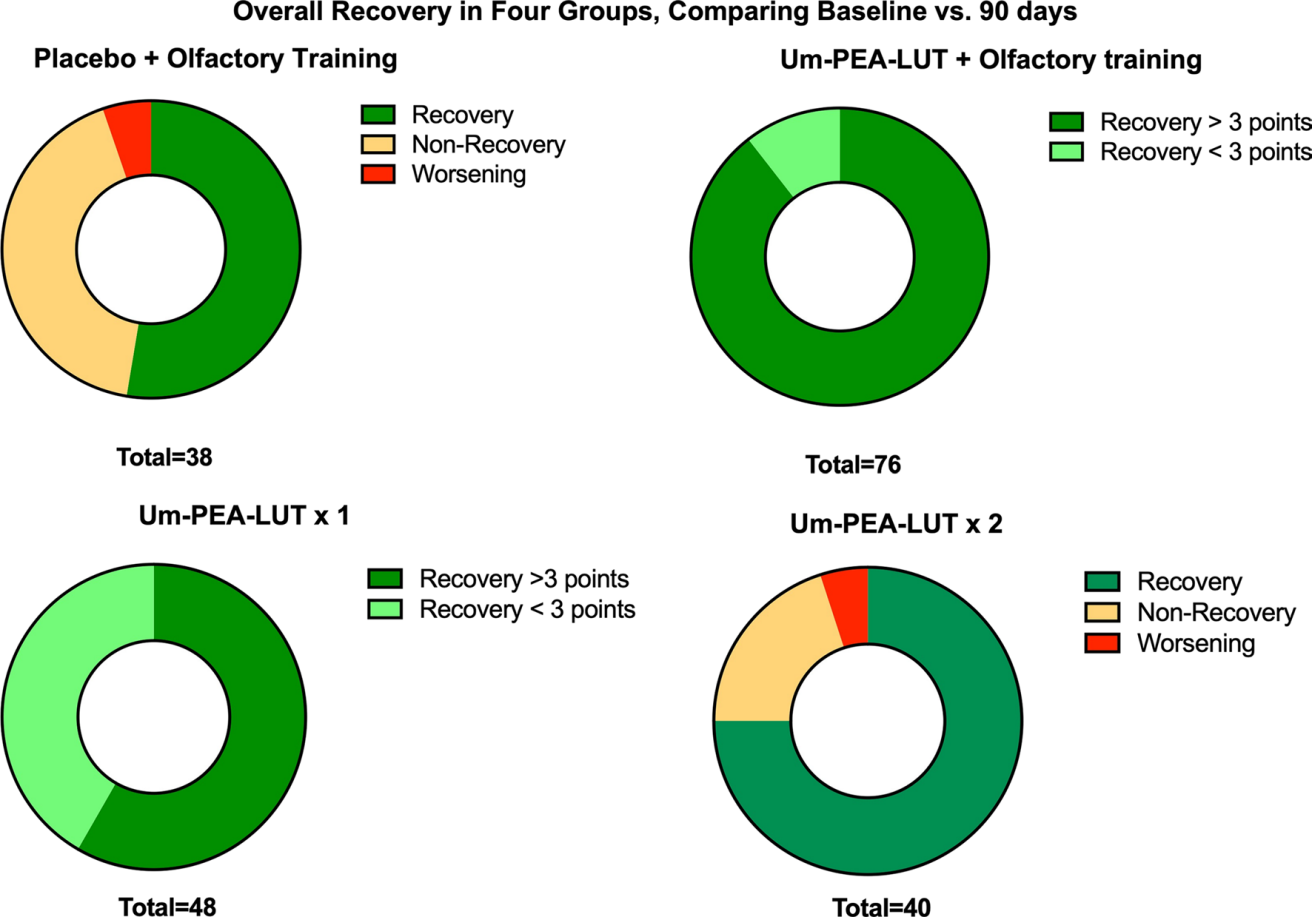
Rings show differences recovery within the four groups. um-PEA–LUT + olfactory training and um-PEA–LUT 1 sachet daily allowed for some recovery of the olfactory functions in 100% patients. *PEA–LUT* ultramicronized palmitoylethanolamide with luteolin

**Fig. 3:**
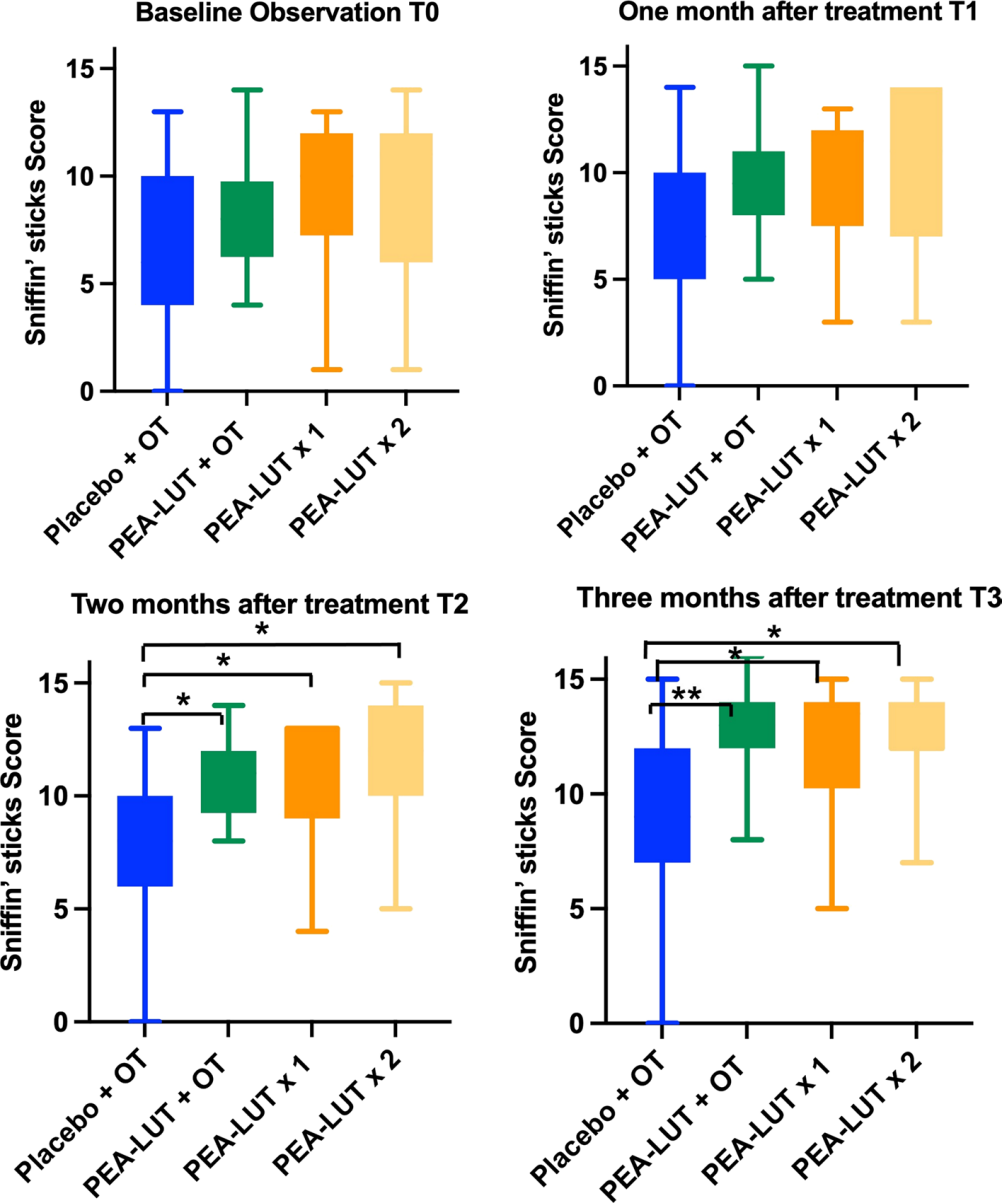
Olfactory scores across groups. Maximal olfactory recovery was observed after 3 months of therapy in the patients who combined um-PEA–LUT and olfactory training. *PEA–LUT* ultramicronized palmitoylethanolamide with luteolin, *OT* olfactory training; “*”*p* < 0.05; “**”*p* < 0.01

When analysis was restricted to improvement ≥ 3 points on Sniffin’ Sticks Score (SSS), combined therapy (um-PEA–LUT + olfactory training group) resulted in significantly more recovery than the other regimens (*χ*: *p* < 0.00001); the analyses of the averages (Table 3) showed statistically significant improvement (ANOVA: *p* < 0.01). Improvements of ≥ 3 points where observed in 89.2% (50 patients; double weighted in randomization) receiving combined therapy group, 41.6% (20 patients) receiving um-PEA–LUT alone—once daily, 40% (16 patients) receiving um-PEA–LUT alone—twice daily, and 36.8% (14 patients) receiving olfactory training plus placebo (Figs. 2, 3). All data were analyzed using the comparative analyses of the SD (Table 3).

**Table 3:** Average and standard deviation of the Sniffin’ sticks scores at the different observation points

Average, SD and CI 95%	*T*_0_	*T*_1_	*p*	*T*_2_	*p*	*T*_3_	*p*	*T*_0_ vs. *T*_3_ *p*
Control	6.5 ± 3.7 (4–11)	7.3 ± 3.4 (4–12)	0.1	8 ± 3.2 (4–13)	0.1	9 ± 3.6 (4–13)	0.1	0.03
PEA–LUT + OT	8.2 ± 2.5 (4–11)	9.9 ± 2.4 (5–13)	< 0.05	10.9 ± 1.8 (8–14)	< 0.01	12.8 ± 1.9 (8–16)	< 0.01	< 0.01
PEA–LUT × 1	9.1 ± 3.4 (2–12)	9.8 ± 2.8 (5–12)	> 0.05	10.9 ± 2.8 (5–13)	> 0.05	12 + 2.8 (5–15)	< 0.01	< 0.01
PEA–LUT × 2	9.6 ± 4.1 (1–13)	9.8 ± 3.9 (5–14)	0.07	11.2 ± 3.1 (5–15)	0.07	12.1 ± 2.2 (7–15)	0.07	< 0.01

The average has been considered including the worsening scores also

*SD* standard deviation, *CI* confidence interval, *PEA–LUT + OT* supplement 1 dose day and olfactory training, *PEA–LUT × 1* supplement 1 dose day, *PEA–LUT × 2* supplement two dose day, *PEA–LUT* ultramicronized palmitoylethanolamide with luteolin, *OT* olfactory training, *SD* standard deviation, *CI* confidence interval

### Secondary outcome: between groups comparison, including subclinical olfactory recovery

When analysis considered any improvement in SSS, to capture subclinical improvements in olfaction, rates of recovery in control group (olfactory training with placebo) were significantly lower than in the three treatment groups receiving um-PEA–LUT (*χ*: *p* < 0.0001). All study participants (100%) who received either um-PEA–LUT + olfactory training or um-PEA–LUT alone—once daily, showed numerical improvement of their SSS. In contrast, in the um-PEA–LUT alone—twice daily group, only 30 (75%) of the 40 patients had some recovery of SSS (Figs. 2, 3). None of the patients suffered from qualitative smell disorders (parosmia, cacosmia).

### Tertiary outcome: within group analysis

Table 3 shows the average and standard deviation of SSS at serial observation points and the results of the statistical analyses performed on these data.

#### Olfactory training plus placebo (multivitamin)

Patients who received placebo plus olfactory training showed no significant improvement in SSS at early timepoints, although improvement was present when comparing SSS at *T*_0_ vs. *T*_3_ (*p* = 0.03). Fourteen (36.8%) of the 38 patients in this group improved their SSS over 3 points at the end of the treatment. Assessing for any change of SSS from *T*_0_ to *T*_3,_ 20 patients (52.6%) improved their SSS, whereas 16 (42.1%) had no change, and 2 (5.3%) had a lower SSS (Fig. 3).

#### um-PEA–LUT alone: once daily

Patients treated with um-PEA–LUT—once daily showed no significant improvement in SSS at early timepoints, although improvement was present when comparing SSS at *T*_0_ vs. *T*_3_ (*p* < 0.01). Twenty patients (41.6%) in this group improved their scores ≥ 3 points when comparing *T*_0_ vs. *T*_3,_ and no patients worsened. Assessing for any numerical change of SSS from *T*_0_ to *T*_3,_ all 48 patients (100%) improved their SSS (Fig. 3).

#### um-PEA–LUT alone: twice daily

Patients treated with um-PEA–LUT alone—twice daily had no significant improvement in SSS at early timepoints, although improvement was present when comparing SSS at *T*_0_ vs. *T*_3_ (*p* < 0.01). Sixteen patients (40%) obtained an improvement of their SSS ≥ 3 points when comparing *T*_0_ vs. *T*_3._ Assessing for any numerical change of SSS from *T*_0_ to *T*_3,_ 30 patients (75%) improved their SSS, 6 (15%) had unchanged SSS, and 4 (10%) had worsening of their SSS (Fig. 3).

#### Combined therapy (um-PEA–LUT + olfactory training)

Patients in the combined therapy group, which received um-PEA–LUT + olfactory training, showed SSS improvement of ≥ 3 points from *T*_0_ vs. *T*_3_ in 89.4% of patients (*p* < 0.0001). At other timepoints, 21.4% (16 patients) improved their SSS ≥ 3 points, 38 (50%) at *T*_0_ vs. *T*_1_; 5 patients (7.1%) improved SSS ≥ 3 points at *T*_2_ vs. *T*_1_; and 24 patients (32.1%) improved their SSS ≥ 3 at *T*_3_ vs. *T*_2_. None of the patients in this group worsen SSS compared to the baseline (*T*_0_). When assessing the for any change of SSS from *T*_0_ to *T*_3,_ all 76 patients (100%) improved their SSS score (Fig. 3).

## Discussion

The nascent literature on persistent COVID-19 olfactory dysfunction underscores the burden of chronic olfactory dysfunction and need for efficacious therapies [30–33]. The findings of our study reaffirm the efficacy of combining um-PEA–LUT with olfactory training, with a ≥ 3 point improvement in nearly 90% of these participants, which was roughly double the rate of recovery in other treatment groups. Although benefit was noted with um-PEA–LUT alone compared to olfactory training alone when considering incremental improvements of 1–2 points in SSS, the clinical significance of the finding is uncertain, given the small magnitude of change.

Although the primary endpoint of this analysis was olfactory recovery at 90 days, the outcomes at sub-intervals were also noted. Study participants receiving um-PEA–LUT + olfactory training regimen demonstrated olfactory recovery of ≥ 3 points by the first follow-up at nearly double the rate (21.4%), of other groups including olfactory training plus placebo (10.4%), um-PEA–LUT alone—twice daily (10%), our um-PEA–LUT alone—once daily (8.3%). Furthermore, at *T*_2_ vs. *T*_3_, SSS recovery ≥ 3 was more common in patients receiving combined therapy (32.1%) than in the other three groups (all < 10%). These data emphasize the synergistic effects of olfactory training and anti-neuroinflammatory therapy [7, 12, 13]. Olfactory training is thought to stimulate the regeneration of olfactory epithelium and to promote restoration of neuronal reconnection [12]. The benefits of combining um-PEA–LUT and olfactory training in this study are consistent with prior reports [13, 14, 19, 34]. We also investigated two dosing schedules to determine whether a once daily vs. twice daily regimen for um-PEA–LUT influenced therapeutic effectiveness or side effects; these outcomes were similar across timepoints, suggesting that once a day dosing of um-PEA–LUT suffices.

Combining um-PEA–LUT supplement with olfactory training was more effective than either olfactory training alone um-PEA–LUT alone. This result could represent either an additive or synergistic effect. The exact mechanisms by which olfactory training works are not fully understood, but olfactory training is thought to induce neuroplastic changes in the olfactory system. Repeatedly exposing the olfactory system to specific odors promotes adaptive changes and rewiring. These changes may involve synaptic strengthening, increased neural connectivity, and the formation of new connections between the olfactory receptor cells and the brain regions involved in olfaction, such as the olfactory bulb and olfactory cortex. Repeated exposure to odorants may help to reactivate and stimulate dormant or weakened olfactory receptors. In terms of odor identification, olfactory training engages individuals to actively focus attention on differentiating and categorizing odors to enhance identification. The active participation and practice inherent involved in olfactory training can also improve attention, concentration, and cognitive processing related to olfaction.

Administering um-PEA–LUT appears to support the neuroplastic changes of olfactory training by fostering a more favorable regenerative milieu. The anti-neuroinflammatory effects of PEA–LUT reduce the inflammation in the olfactory bulbs and allow normal growth of immature neurons [35]. In the context of persistent post-COVID-19 anosmia, um-PEA–LUT could facilitate the re-growth between olfactory neurons and glomeruli; the reduction of the inflammation creates a supportive environment for normal synaptic reconnection. Ultramicronization of um-PEA–LUT increases bioavailability. Once absorbed, it can reduce mastocyte degranulation and proliferation [36], reduce reactive oxygen species, and promote polarization of microglia from M1 state (inflammatory and neurotoxic) to M2 state (anti-inflammatory and neuroprotective) [37, 38]. SARS-CoV-2 infection induces microglial state changes in the brains of patients with COVID [39], so in patients receiving um-PEA–LUT, lutein-induced reduction of reactive oxygen species [40] and PEA-related polarization of microglia [41] likely both contribute to improved response to olfactory training.

Reduced inflammation in the peripheral neuroepithelium and olfactory bulbs is conducive to recovery of connections during olfactory training. Further work might explore whether um-PEA–LUT might alleviate other neuroinflammation-related symptoms, including headache, anxiety, cough, or impaired cognition [12, 14, 42, 43]. Preliminary work suggests that um-PEA–LUT might alleviate brain fog, consistent with the known link between olfactory function and memory [14, 23, 44].

It is important to comment on patterns of attrition. We observed 24% of attrition both in the placebo + OT group and in the um-PEA–LUT + OT group. However, despite similar rates of attrition, the attrition seemed attributable to distinct reasons. In the case of the Placebo + OT group, the patients who did not present to all follow-up visits explained their missed appointment as not observing improvement (in several cases these patients presented at baseline and at the end of study). On the contrary, patients in the um-PEA–LUT reported that they did not present to all follow-ups (mostly arrived at the second only), because they recovered before the end of the study. In the um-PEA–LUT twice-daily group, some patients reported dropping out of the study, because they experienced a swift recovery of the olfaction after a single month of use, whereas um-PEA–LUT once a day group might have had more gradual recovery. These explanations of patients are anecdotal, however, and further studies are needed; the lower attrition in the once daily group receiving um-PEA–LUT may also reflect the relative ease of a once daily dosing regimen without olfactory training.

### Study Limitations

This study has limitations relating to enrollment, interventions, and assessment. The population affected by olfactory loss and other manifestations of long-COVID is heterogeneous, variously suffering from fatigue, cognitive, and respiratory symptom clusters [45]. Although our study achieved its target enrollment, there was some variance in number of patients allocated per group, gender distribution, and baseline olfactory data (differences not statistically significant). Risk of bias was minimized through randomization, blinding, and consistent procedures; but we did not placebo-control for once daily vs. twice daily dosing. All patients received therapy (an untreated control group would not have been equipoise), and spontaneous improvement may have occurred. Although we incorporated regular patient check-ins, we could not verify adherence to the treatments by direct observation, since the olfactory training and supplements occurred at home.

The weighted randomization scheme increased exposure of participants to a putative gold standard, but this approach contributed to smaller size of remaining groups and modest reduction in statistical power; the failure to detect any significant demographic associations should be interpreted accordingly. The analysis also did not incorporate serological or radiological biomarkers to assess changes in neuroinflammation or pharmacokinetic analysis, both are areas for future research.

Nearly 1 in 5 of participants enrolled in this study dropped out before completion. This attrition introduces a risk of bias, since individuals who dropped out of the study might differ from those who completed the study. Our analysis did not find differences in baseline olfactory function or response to therapy between those who dropped out vs. those who completed the study. However, there was a lower rate of attrition in the once daily um-PEA–LUT alone group compared to other groups. Taking a supplement once a day is less burdensome than taking a supplement twice daily or combining the supplement olfactory training, which may partly explain this finding. We did not systematically collect data on reasons for dropout from the study. Possible contributors include logistical hurdles of the trial, impaired functional status from long COVID, perceived lack of benefit, perceived lack of need to complete the study if recovered, or interactions between these factors.

Olfactory outcomes were performed with the validated Sniffin’ Sticks test using odor identification measures to maximize feasibility of serial assessments, consistent with prior work showing only a small effect of olfactory training on the threshold subtest for odor detection [46]. We defined chronic olfactory loss as > 6 months prior to enrollment to ensure adequate washout, so the experimental timeline spanned 3 months; studies in neurodegenerative disorders with um-PEA–LUT often span greater than 6 months, and different results might be observed over longer time horizons. The number of patients enrolled exceeded the minimum of 200 defined in the registered clinical trial; the study centers varied in number of patients recruited (different geographical distribution of patients affected by long-COVID), and this variance may have contributed to imbalance within groups. In addition, the study did not identify instances of parosmia, which are known to occur in a subset of patients.

In assessing olfactory recovery, the analysis was limited to use of “I” (odor identification) instead of the full TDI battery (odor threshold, identification, and discrimination), which was a deviation from the original clinical trial protocol. This modification resulted in less comprehensive analysis than the full TDI battery, and the rigor of odor identification in our study falls short of the internationally accepted standard of the University of Pennsylvania Smell Identification Test (UPSIT), which offers extensive normative data and a broad range of 40 or more odorants spanning a wide spectrum of olfactory stimuli. In the long-COVID population, which often experiences brain fog or other cognitive manifestations, participant fatigue and sustained attention were significant concerns. The abridged assessment based on identification is limiting and relied on use of clinically significant difference of three points compared to a difference of five points for the full TDI Battery; we cannot assess whether the single treatment with um-PEA–LUT might improve threshold or detection. Additional studies using TDI or UPSIT are needed to corroborate findings and determine generalizability to larger populations. In addition, limitations relating to blinding, randomization, and attrition increased risk of bias. Assignment to placebo vs. supplement was blinded for patients, clinicians, and statistician, but there was no patient blinding for once-daily vs. twice-daily supplement or for whether olfactory training was performed. In addition, a significant portion of patients enrolled did not complete the study, contributing to imbalance between the experimental groups in gender distribution and olfactory function.

## Conclusion

The results of this study supports the efficacy of combining olfactory training with um-PEA–LUT for recovery from persistent COVID-19 olfactory dysfunction. Further prospective studies incorporating pharmacokinetic assessment and biomarker analyses are necessary to determine distribution in neural tissues affected by SARS-CoV-2 and to evaluate anti-neuroinflammatory phenomena. Additional studies are also necessary to assess long-term outcomes and to further refine regimens for treating persistent olfactory dysfunction and other manifestations of long COVID.

## Data Availability

The original data are available under request to corresponding author.
